# The Impact of Neighborhood-level Racial and Economic Segregation on Low-Risk Cesarean Delivery among Black, White, and Biracial (Black/White) Individuals

**DOI:** 10.21203/rs.3.rs-7643886/v1

**Published:** 2025-10-09

**Authors:** Ella Batterson, Shira Goldenberg, Rebecca J. Baer, Gretchen Bandoli

**Affiliations:** San Diego State University; San Diego State University; University of California, San Diego; University of California, San Diego

**Keywords:** cesarean, racism, segregation, Black, White, biracial, multiracial, maternal, disparity, Index of the concentration at the Extremes

## Abstract

**Background:**

Research has established Black-White low-risk cesarean delivery (CD) disparities; however, it is unknown how select structural factors are involved in this disparity and whether Biracial (Black/White) individuals face similar disparities. Our objective was to estimate the association of low-risk CD among Black, White and Biracial individuals, and determine whether these associations vary by neighborhood level racial and economic segregation.

**Methods:**

385,825 nulliparous, term, singleton, vertex births among Black, White, and Biracial individuals in California (2011–2019) were included from a statewide administrative birth cohort of birth certificates linked to hospital records. We used a generalized estimating equation, Poisson regression stratified by Index of the Concentration of the Extremes (ICE) tertile to estimate risk ratios (RR) for low-risk CD across tertiles of racial and ethnic disparities. The Index of the Concentration at the Extremes (ICE; American Community Survey) is a measure of racial and economic segregation where ICE tertiles 1–3 rank census tracts from most to least impacted by inequality. Models were adjusted for maternal age at delivery. We also assessed the potential mediating roles of socioeconomic factors, maternal characteristics, and quality of care variables through regression-based mediation analyses.

**Results:**

The risk of CD was greatest in Black individuals (30.71%), followed by Biracial (25.47%) and White (24.98%). In age adjusted models, Black individuals had a higher CD risk than White individuals across all tertiles, with similar estimates within racial and economic segregation tertile (aRR_tertile1_: 1.34; 95% CI: 1.21, 1.36, aRR_tertile2_: 1.35; 95% CI: 1.30, 1.39, aRR_tertile3_: 1.40; 95% CI: 1.33, 1.47). Biracial individuals had a higher risk for CD than White individuals in all tertiles after age adjustment (aRR_tertile1_: 1.16; 95% CI: 1.10, 1.22, aRR_tertile2_: 1.18; 95% CI: 1.10, 1.27, aRR_tertile3_: 1.18; 95% CI: 1.08, 1.29). Select socioeconomic factors and maternal characteristics were identified as mediators.

**Conclusions:**

The low-risk CD disparity by race persisted across all ICE tertiles. Biracial individuals experienced a higher risk of CD than White, but not Black individuals suggesting that they may experience simultaneous health advantages and disadvantages relative to their monoracial counterparts.

## Background

Low-risk cesarean delivery (CD) refers to nulliparous, term, singleton, and vertex (NTSV) births through cesarean sections. Cesarean deliveries can be lifesaving procedures when necessary, but they are major surgical interventions that can lead to adverse outcomes (1), including future subfertility and subsequent pregnancy risks such as placenta previa, uterine rupture, and stillbirth (2). Given the risks associated with CD, Healthy People 2030 aims to decrease the low-risk CD rate to 23.6%. In 2019, California was able to achieve a low-risk CD rate of 22.8%; however, progress has been unequal and racial and ethnic disparities remain (3). Among low-risk individuals in California, Black individuals have 1.3 times the risk of a CD compared to White individuals after adjusting for clinical conditions (4). Between 2011–2017, White individuals saw an 11% decrease in their low-risk CD rate, compared to a 1% decrease among Black individuals (4). The unchanged CD disparity suggests that new interventions and policy changes are still needed to reach maternal health equity.

Structural racism refers to the large-scale systems, social forces, institutions, ideologies, and processes that interact to create and perpetuate inequalities among racial and ethnic groups. (5). Segregation measures are a common indicator of structural racism due to historical redlining policies that led to disinvestment in predominantly Black communities (6, 7). Research has shown that aspects of structural racism measured through racial, economic, and residential segregation are associated with chronic health conditions and psychosocial factors that increase their risk for pregnancy-related complications (8–12). In particular, Black individuals are more likely to have risk factors for CD, such as high body mass index (BMI) and diabetes, which are hypothesized to be due to life course exposure to racism, sexism, and social and economic inequality (13). A 2024 study in California using a neighborhood deprivation index reported that Black individuals had a higher risk of CD at all levels of socioeconomic disadvantage compared to White individuals (14). The Index of the Concentration at the Extremes (ICE) score is a popular measure that accounts for spatial and economic polarization simultaneously, which helps decrease multicollinearity issues (7). The ICE measure has demonstrated that social and economic segregation, as a proxy for structural racism, is associated with severe maternal morbidity (SMM) and preterm birth among Black individuals, but this measure has not been used to investigate the CD disparity (6, 15, 16).

While most maternal health research has examined Black-White disparities, there is a growing multiracial population in the United States, with the White and Black-identifying population growing by 67.4% from 2010 to 2020 (17). Most health research has excluded the Biracial population and the lasting impacts of the “one drop rule” used to categorize anyone with “Black blood” as Black (18). Yet, a growing body of research supports disaggregation of the multiracial group, as differences in health outcomes have been revealed when specific subgroups of Biracial people are compared (19). Furthermore, a study comparing the risk of preterm birth among those who identified as Black and White revealed that they had a lower risk of preterm birth than their Black counterparts, but a higher risk than their White counterparts (20). Since CD disparities are most pronounced when comparing Black to White individuals, examining the Biracial-White disparity will provide actionable evidence in this understudied population.

While we are aware of low-risk CD disparities among Black individuals, to our knowledge, there has been no published research examining the CD rates among Biracial groups, nor how racial and economic segregation could play a role in the low-risk CD disparity. Using a statewide birth cohort from California between 2011–2019, we examined whether racial disparities in low-risk CD are modified by a measure of racial and economic segregation in a population of Black, White, and Biracial individuals. We hypothesized that the risk of CD would be highest among Black individuals compared to Biracial and White individuals, and that the racial disparity would be largest in areas that experience the most segregation, with Biracial individuals having a higher risk of CD than White individuals having a higher relative and absolute risk of CD than White individuals within areas that experience the least segregation. We use the term “Biracial” to refer to those who identify as both Black and White and “individuals” to refer to people who gave birth.

## Methods

### Study design and population

The sample was drawn from the Study of Mothers and Infants (SOMI), an administrative birth cohort consisting of individuals who delivered liveborn infants in California between 2005–2021. To create the cohort, birth certificates were probabilistically linked to hospital, emergency department, and ambulatory surgery records from the Department of Health Care Access and Information (HCAI) for both mothers and infants, covering the period from one year before birth through one year after birth (21). These linked records included diagnostic and procedure codes based on the International Classification of Diseases, 9th Revision (ICD-9), and 10th Revision, Clinical Modification (ICD-10) reported to HCAI by the hospitals. The SOMI was approved by the University of California, San Diego (UCSD) and the Committee for the Protection of Human Subjects within the Health and Human Services Agency of the State of California (21).

**Figure 1.**
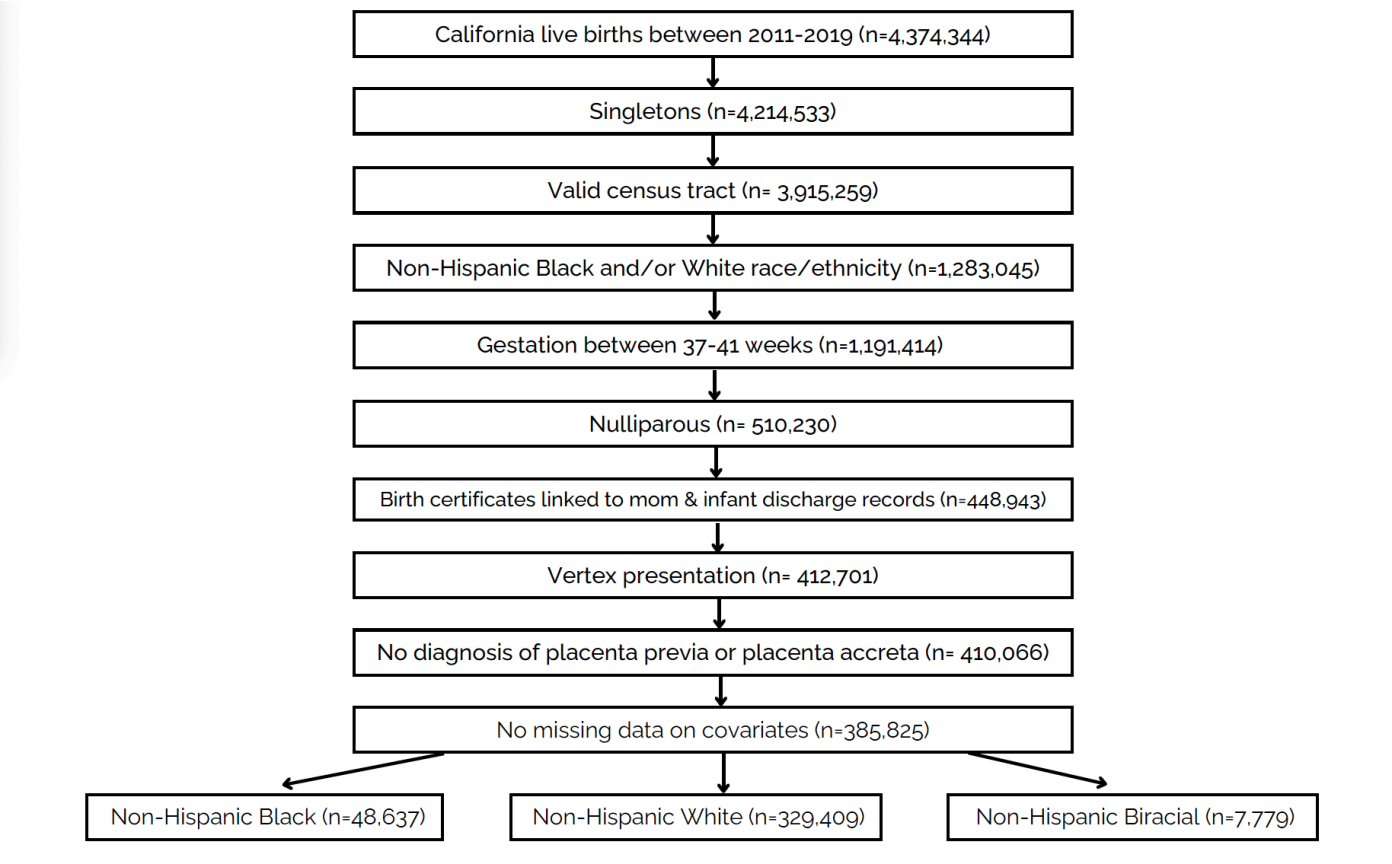
Sample selection figure: California 2011–2019

The sample was restricted to nulliparous, term (37– 41week gestation) singleton vertex births delivered between 2011–2019 to women who identified as non-Hispanic Black, White, or Biracial. Additional inclusion criteria included a valid residence census tract and successful linkage of birth certificates and hospital discharge records for mother and infant. Individuals that had a fetus in nonvertex presentation and those with placenta previa or placenta accreta were excluded. Finally, we excluded those with missing data on BMI, education, adequacy of prenatal care, mode of delivery, age, primary attendant at birth, gestational size, and payer source for delivery, resulting in a final sample of 385,825 (Fig. 1).

### Maternal Characteristics

Maternal characteristics were selected to describe the sample based on a literature review and data availability from birth certificates (1, 4–6, 10, 12, 17, 24, 25). The characteristics included maternal age at delivery (years), maternal education (less than high school, high school degree or equivalent, some college, and college degree), insurance type (private, public, other), Special Supplemental Nutrition Program for Women, Infants, and Children (WIC) enrollment (yes, no, missing), rurality (urban, rural), gestational age at delivery (37–41 weeks), induction of labor (yes, no), body mass index (BMI, kg/m2, calculated from pre-pregnancy weight and height), primary attendant at birth (midwife, M.D/D.O, other), and birthweight for gestational age (SGA, AGA, LGA). We assessed adequacy of prenatal care (adequate, intermediate, and inadequate), using the Adequacy of Prenatal Care Utilization (APNCU) index (22). Hypertension and diabetes (pregestational or gestational) were obtained from maternal HCAI records and birth records. The data sources for outcomes, variables, and relevant ICD codes are provided in Supplemental Table 1.

### Measurement of Exposure & Outcome

Self-reported race and ethnicity were obtained from birth records. The outcome of cesarean delivery was also abstracted from birth records from diagnoses and procedure codes based on the ICD-9 and ICD-10 as reported to the California Office of Statewide Health Planning and Development by the hospitals. The low-risk CD criteria was consistent with prior research and included nulliparous, term (37– 41week gestation), singleton, and vertex births without placenta previa or placenta accreta (4, 14).

### Measurement of ICE tertiles

We linked the data with census tract annual 5-year estimate data available from the ACS, 2011–2019 to generate ICE scores by census tract for each individual. The ICE_Race−Income_ score is a multidimensional measure used to examine racial and economic segregation (23). The formula is defined as ICE*i* = (A*i*-P*i*)/T*i* where *i* can be race, income, or race and income. The ICE_Race−Income_ measure combines data on income and race. A*i* represents the privileged extreme consisting of white residents in the 80th income percentile (>$100,000) and P*i* represents the deprived extreme consisting of Black residents in the 20th income percentile (<$25,000) in a given area (23). The score assigns subjects a score from − 1 (most disadvantaged) to 1 (most advantaged) based on the area they live to calculate the directions of racial economic segregation (13). The ICE_Race−Income_ score was calculated for each census tract using ACS annual 5-year estimates. A score was assigned to everyone in this analysis to represent the degree of racial and economic segregation based on where they live. ICE_Race−Income_ scores were categorized into three tertiles based on the study sample distribution with tertile one consisting of individuals living in census tracts with the highest concentration of low-income Black residents and tertile 3 consisting of the highest concentration of high-income White residents.

### Mediator Variables

Based on Directed Acyclic Graphs, all covariates were conceptualized as potential as mediators given the lack of causal pathways between measured variables and our primary exposures of race/ethnicity. The mediators included socioeconomic factors measured through education, rurality, insurance type, and WIC enrollment. Quality of care was measured through adequacy of prenatal care, primary attendant for birth, and induction of labor. Maternal characteristics included hypertension, diabetes, and pre-pregnancy BMI (underweight [< 18.5 kg/m2], normal [18.5–24.9 kg/m2], overweight [25.0–29.9 kg/m2], and obese [30.0 kg/m2]).

### Statistical Analysis

To test the relationship between race/ethnicity and CD we used Generalized Estimation Equation (GEE) Poisson regression to account for clustering by census tract. Models were stratified by ICE_Race−Income_ tertile to examine whether associations between race/ethnicity and CD differ by racial and economic segregation. Within each tertile, non-Hispanic White individuals served as the reference. We produced models to calculate both risk ratios and risk differences to estimate the relative and absolute effects of race/ethnicity on CD stratified by ICE_Race−Income_. The first set of models were unadjusted and the second were age-adjusted. While by definition, age is not a confounder, we adjusted for it due to its well-documented association with CD and its imbalance across racial groups (24).

### Mediation Analysis

Finally, we conducted a decomposition analysis to assess the role of mediating factors between race/ethnicity and CD within the same ICE tertile. We used the SAS macro% *mediation* to obtain the controlled direct effect, natural indirect effect, and proportion mediated by each mediator variable (25). Selected mediators included education, insurance type, primary attendant at birth, rurality, WIC enrollment, BMI, induction of labor, adequacy of prenatal care, hypertension, and diabetes.

## Results

### Descriptive results

The sample consisted of 385,825 low-risk births, among which 329,409 (85.4%) were Non-Hispanic White, 48,637 (12.6%) were Non-Hispanic Black, and 7,779 (2.0%) were Non-Hispanic Biracial birthing people [Table 1]. Most of the Black population lived in the areas experiencing the highest racial and economic segregation that consist of predominantly Black and low-income residents (tertile 1). Although less than the Black population, most of the Biracial population (53.5%) also lived in these highly disadvantaged areas whereas the White population predominantly lived in tertiles 2 and 3 (73.9%).

Compared with the White population, the Black and Biracial populations had a younger median age at delivery (25 vs 29 years), were more likely to be obese (Black: 25.8%; Biracial: 24.0%, White: 15.0%), and receive inadequate prenatal care (Black: 13.9%; Biracial: 11.9%, White: 6.2%;). The prevalence of public insurance, WIC enrollment, and small birthweight for gestational age among Biracial individuals fell in between those of Black and White individuals. Compared with Black individuals, the Biracial population was more similar to the White population in terms of living in a rural area and being diagnosed with preeclampsia.

### Regression results

The highest prevalence of low-risk CD was in Black individuals (30.7%), followed by Biracial (25.4%) and White individuals (24.9%) [Table 1]. There was little heterogeneity in this risk by ICE tercile, with the exception of a slightly lower risk among White individuals from terciles 1 to 3 [Table 2]. Black individuals had a consistent increased risk for CD compared to White individuals across all tertiles, although estimates were fairly similar across tertiles (aRR_tertile1_: 1.34; 95% CI: 1.21, 1.36, aRR_tertile2_: 1.35; 95% CI: 1.30, 1.39, aRR_tertile3_: 1.40; 95% CI: 1.33, 1.47). After age adjustment, Biracial individuals had an increased risk for CD compared to White individuals in all tertiles, but lower than Black individuals (aRR_tertile1_: 1.16; 95% CI: 1.10, 1.22, aRR_tertile2_: 1.18; 95% CI: 1.10, 1.27, aRR_tertile3_: 1.18; 95% CI: 1.08, 1.29). Among Biracial individuals, there was no heterogeneity in CD risk estimates by ICE tercile. On the absolute scale, the difference in the risk of CD between Black and White individuals was also consistent across tertiles, and the disparity was largest in tertile 3 (aRD_tertile1_: 7.63%; 95% CI: 7.07%, 8.19%, aRD_tertile2_: 7.57%; 95% CI: 6.60%, 8.53%, aRD_tertile3_: 8.79%; 95% CI: 7.23%, 10.35%). The difference in the risk of CD between Biracial and White individuals increased after age adjustment (aRD_tertile1_: 3.69%; 95% CI: 2.35%, 5.03%, aRD_tertile2_: 4.05%; 95% CI: 2.26%, 5.84%, aRD_tertile3_: 4.09%; 95% CI: 1.83%, 6.35%).

### Mediation results

In mediation analyses comparing Black and White individuals, being overweight or obese (BMI 25+) was the most substantial mediator (tertile 1: 19.00%, tertile 2: 21.13%, tertile 3: 21.93%) [Table 3]. WIC enrollment mediated more of the low-risk CD disparity in areas with more racial and economic segregation (tertile 1: 16.57%, tertile 2: 11.98%, tertile 3: 6.47%), whereas having public insurance mediated approximately 12% in all tertiles. Hypertension contributed to a small part of the low-risk CD disparity, with a greater effect in tertile 1 (tertile 1: 7.10%, tertile 2: 4.94%, tertile 3: 3.32%). The same patterns were observed in the mediation analyses comparing Biracial to White individuals. Having a BMI ≥ 25 was the most substantial mediator (tertile 1: 27.49%, tertile 2: 35.28%, tertile 3: 26.19%), and WIC enrollment mediated more of the disparity among Biracial individuals compared to Black individuals (tertile 1: 31.68%, tertile 2: 17.68%, tertile 3: 13.10%) [Table 4]. Primary attendant at birth, induction, adequacy of prenatal care, rurality, and diabetes did not act as mediators.

## Discussion

In this study we found differences in low-risk CD among Black, White, and Biracial individuals which provides insight on maternal health equity. The prevalence of CD was greatest in Black individuals (30.71%), followed by Biracial (25.47%) and White (24.98%) individuals. The prevalence is slightly lower compared to other studies which is likely due to the decrease in CD incidence in more recent years (4,14). When examining whether differences in CD varied by census-level racial and economic segregation, we found that Black individuals had an increased risk of CD compared to White individuals in all tertiles, but there was little difference is risk estimates across tertiles. This finding was consistent with another study investigating low-risk CD among California individuals using a neighborhood deprivation index, which found that Black individuals had an increased risk of CD compared to White individuals at all levels of advantage (14). Despite overlapping confidence intervals across tertiles, we observed a very modest increase in the disparity with decreasing racial and economic segregation, with the highest risk disparities occurring in areas with the least racial and economic segregation. This differed from our hypothesis that the racial disparity would be largest in the areas that experience the most structural racism, and the results appear to be due to the slight decline in prevalence of low-risk CD among White individuals in areas of less racial and economic segregation, but not among Black and Biracial individuals.

This study was novel in its examination of potential mediators that could explain the observed low-risk CD disparity by race/ethnicity. The greater mediating effect of WIC enrollment on the low-risk cesarean delivery disparity in tertiles 1 and 2 supports its role as a proxy for income status. This suggests that a larger proportion of Black and Biracial individuals may experience low-income status and food insecurity than White individuals, contributing to the low-risk CD disparity. We also found that being overweight or obese mediated 19 – 22% of the Black-White low-risk CD disparity across tertiles. In this analysis, Black individuals had a higher prevalence of obesity and hypertension than White individuals, which is a known risk factor for CD along with gestational hypertension and diabetes (26, 27).

### Policy and Program Recommendations

The lack of substantial variation in the low-risk CD disparity by racial and economic segregation, as well as the relatively modest contribution of mediators, suggests that future intervention efforts need to use data sources that capture a larger and more contextual framework of risk factors. However, the persistent racial disparities noted across studies call for more urgent actions. Clinicians’ education should include continuous cultural competency training that addresses implicit biases and shows how to create a supportive relationship with their patients (28). Research has revealed that Black individuals who had a CD in their first pregnancy have low rates of shared decision making with their providers (29). CD rates are consistently lower in birth centers that follow models involving prenatal care around birth preparation, relationship building between the patient and midwife, and shared decision making (30). Shifting typical hospital care to focus on building patient relationships, shared decision making, and increasing opportunities for racially concordant care can improve the maternal experience, promote agency, and increase health literacy (31, 32). Implementing a standardized labor induction protocol at hospitals is also associated with a decreased CD rate among Black individuals, indicating that having clear labor management guidelines can mitigate the potential for implicit bias from physicians in the cesarean delivery decision process (27). While these findings need confirmation, our data suggest that obesity prevention could be a beneficial pathway for public health interventions among Black and Biracial populations to reduce the CD rate and promote maternal health. In addition, Black and Biracial individuals enrolled in WIC may be an important priority population for maternal health interventions to reduce stress and improve healthy food access before, during, and after pregnancy.

### Limitations and Strengths

Strengths of this study include its large sample and ability to include the Biracial population. Additionally, to better inform public health practices, we calculated both relative and absolute risks and performed a mediation analysis instead of adjusting for maternal characteristics to avoid blocking pathways that can be evaluated as intervention targets. The ICE_Race-Income_ score allowed us to capture the collective impact of census tract level racial and economic segregation on the low-risk CD disparity; However, it does not recognize the extent of travel between neighborhoods, individual level exposures to racism, or residential mobility during pregnancy. Due to ACS data limitations, the score was modeled to include only Black and White individuals and was not able to include Biracial individuals. We also relied on administrative data which may fail to capture the reasons for CD and other factors that influence the disparity.

## Conclusion

In this California cohort, the Black-White low-risk CD disparity persisted regardless of racial and economic segregation. Biracial individuals experienced a higher risk of CD than White individuals and lower risk than Black individuals for CD suggesting that they experience simultaneous advantages and disadvantages to their monoracial counterparts. Further research should examine the role of indications for CD, and provider characteristics such as their training, bias, race/ethnicity, and gender in the CD disparity. Additionally, a qualitative assessment of an individual’s experiences in the hospital during labor could contextualize the persistent disparity in CD by race. Finally, further research is recommended to understand the potentially unique experiences of Biracial populations.

## Supplementary Material

Supplementary Files

This is a list of supplementary files associated with this preprint. Click to download.

• ManuscriptSupplementalMaterial.docx

## Figures and Tables

**Table 1.** Characteristics of low-risk births, overall and by race/ethnicity, California 2011–2019 (n = 385,825)

Race/Ethnicity				
	Total n=385,825 (%)	Non-Hispanic White n=329,409 (%)	Non-Hispanic Black n=48,637 (%)	Non-Hispanic Biracial^1^ n=7,779 (%)
**Mode of delivery**				
Cesarean	99,212 (25.71)	82,297 (24.98)	14,934 (30.71)	1,981 (25.47)
**ICE Race-Income** ^2^				
Tertile 1	126,474 (32.78)	85,971 (26.10)	36,344 (74.73)	4,159 (53.46)
Tertile 2	131,770 (34.15)	120,526 (36.59)	8,979 (18.46)	2,265 (29.23)
Tertile 3	127,581 (33.07)	122,912 (37.31)	3,314 (6.81)	1,335 (17.42)
**Age at delivery**				
Median (IQR)	29 (20–38)	29 (21–37)	25 (16–34)	25 (16–34)
35 or more years	60,191 (15.60)	55,783 (16.93)	3,820 (7.85)	588 (7.56)
**Education**				
Less than high school	17,631 (4.50)	10,448 (3.17)	5,991 (12.32)	922 (11.85)
High school or equivalent	72,6109 (18.82)	54,474 (16.54)	15,890 (32.67)	2,246 (28.87)
Some college	77,625 (20.12)	61,308 (18.61)	14,022 (28.83)	2,295 (29.50)
College degree	218,229 (56.56)	203,179 (61.68)	12,734 (26.18)	2,316 (29.77)
**Insurance type**				
Private	274,291 (71.09)	250,057 (75.91)	20,236 (41.61)	3,998 (51.39)
Public	99,920 (25.90)	69,422 (21.07)	26,916 (55.34)	3,582 (46.05)
Other	11,614 (3.01)	9,930 (3.01)	1,485 (3.05)	199 (2.56)
**WIC participation**				
Yes	104,298 (27.03)	67,941 (20.63)	32,210 (66.23)	4,147 (53.31)
No	279,721 (72.50)	259,841 (78.88)	16,277 (33.47)	3,603 (46.32)
Unknown	1,806 (0.47)	1,627 (0.49)	150 (0.31)	29 (0.37)
**Rurality**				
Urban	289,341 (74.99)	241,940 (73.45)	41,483 (85.30)	5,918 (76.08)
Rural	96,484 (25.01)	87,469 (26.55)	7,154 (14.70)	1,861 (23.92)
**Adequacy of prenatal care**				
Inadequate	28,222 (7.31)	20,507 (6.23)	6,792 (13.96)	923 (11.87)
Intermediate	58,202 (15.09)	47,714 (14.48)	9,100 (18.71)	1,388 (17.84)
Adequate	299,401 (77.60)	261,188 (79.29)	32,745 (67.33)	5,468 (70.29)
**Gestational age at delivery**				
37th week	24,844 (6.44)	20,280 (6.16)	4,080 (8.39)	484 (6.22)
38th week	52,607 (13.63)	43,400 (13.18)	8,059 (16.57)	1,148 (14.76)
39th week	121,592 (31.51)	103,235 (31.34)	15,827 (32.54)	2,530 (32.52)
40th week	127,687 (33.09)	110,322 (33.49)	14,829 (30.49)	2,536 (32.60)
41st week	59,095 (15.32)	52,172 (15.84)	5,842 (12.01)	1,081 (13.90)
**Induction of labor**	214,272 (55.54)	184,267 (55.94)	25,770 (52.98)	4,235 (54.44)
**Prepregnancy BMI (kg/m2)**				
Underweight (<18.5)	16,989 (4.40)	14,286 (4.34)	2,321 (4.77)	382 (4.91)
Normal (18.5–25)	218,121 (56.53)	191,303 (58.62)	21,378 (43.95)	3,640 (46.79)
Overweight (25–29.9)	86,691 (22.47)	72,402 (21.98)	12,398 (25.49)	1,891 (24.31)
Obese (≥30)	64,024 (16.59)	49,618 (15.06)	12,540 (25.78)	1,866 (23.99)
**Primary attendant for birth**				
M.D./D.O.	335,780 (87.03)	286,926 (87.10)	42,166 (86.70)	6,688 (85.98)
Midwife^3^	49,303 (12.78)	41,855 (12.71)	6,372 (13.10)	1,076 (13.83)
Other	742 (0.19)	628 (0.19)	99 (0.20)	15 (0.19)
**Birthweight for gestational age**				
SGA	37,298 (9.67)	28,807 (8.50)	8,344 (17.16)	947 (12.17)
AGA	316,226 (81.96)	272,051 (82.59)	37,923 (77.97)	6,252 (80.37)
LGA	32,301 (8.37)	29,351 (8.91)	2,370 (4.87)	580 (7.46)
**Comorbidities**				
Any hypertension^4^	46,993 (12.18)	38,206 (11.60)	7,776 (15.99)	1,011 (13.00)
Pregestational	5,857 (1.52)	4,723 (1.43)	1,016 (2.09)	118 (1.52)
Gestational	20,297 (5.26)	17,033 (5.17)	2,822 (5.80)	442 (5.68)
Preeclampsia	18,195 (4.72)	14,305 (4.34)	3,496 (7.19)	394 (5.06)
Any diabetes	26,888 (6.97)	23,377 (7.10)	3,097 (6.37)	414 (5.32)
Pregestational	2,521 (0.65)	2,060 (0.63)	412 (0.85)	49 (0.63)
Gestational	24,367 (6.32)	21,317 (6.47)	2,685 (5.52)	365 (4.69)

Abbreviations: WIC, The Special Supplemental Nutrition Program for Women, Infants, and Children; BMI, body mass index; M.D., Doctor of Medicine; D.O. Doctor of Osteopathic Medicine; SGA, small for gestational age; AGA, appropriate for gestational age; LGA, large for gestational age

1 Biracial refers to individuals who identified as two races: Black and White

2 ICE_Race-Income_ is the relative concentration of low-income black households vs. high income white households in mothers’ ZIP code of residence; tertile one is the highest relative concentration of low-income black households, and tertile three is the lowest.

3 A midwife includes a certified nurse midwife, licensed midwife, or other midwife

4 The type of diagnosed hypertension was not always specified, leaving more individuals included in the “any hypertension” category than the subtypes

**Table 2.** Association between race/ethnicity and low-risk CD by ICE_Race-Income_, California 2011–2019

		%	RD^1^ (95% CI)	aRD (95% CI)	RR (95% CI)	aRR (95% CI)
**Decreasing segregation**	**Tertile 1**					
	White	25.76	reference	reference	reference	reference
	Black	30.67	4.91 (4.36, 5.47)	7.63 (7.07, 8.19)	1.19 (1.17, 1.21)	1.34 (1.21, 1.36)
	Biracial	25.53	−0.23 (−1.59, 1.13)	3.69 (2.35, 5.03)	0.99 (0.94, 1.05)	1.16 (1.10,1.22)
	**Tertile 2**					
	White	25.11	reference	reference	reference	reference
	Black	30.73	5.62 (4.63, 6.60)	7.57 (6.60, 8.53)	1.22 (1.18, 1.26)	1.35 (1.30, 1.39)
	Biracial	25.56	0.45 (−1.36, 2.26)	4.05 (2.26, 5.84)	1.02 (0.95, 1.09)	1.18 (1.10, 1.27)
	**Tertile 3**					
	White	24.31	reference	reference	reference	reference
	Black	30.99	6.66 (5.07, 8.26)	8.79 (7.23, 10.35)	1.27 (1.21, 1.34)	1.40 (1.33, 1.47)
	Biracial	25.09	0.76 (−1.56, 3.08)	4.09 (1.83, 6.35)	1.03 (0.94, 1.13)	1.18 (1.08, 1.29)

Abbreviations: CD, cesarean delivery

1 Risk differences are presented as cases of cesarean per 100 live NTSV births

**Table 3.** Age-adjusted mediation results for low-risk CD comparing Black to White individuals by ICE_Race-Income_, California 2011–2019.

	Tertile 1 (most segregated)	Tertile 2	Tertile 3 (least segregated)
**High School or Less vs Some College or Higher**			
Direct	1.32 (1.29, 1.35)	1.33 (1.28, 1.39)	1.38 (1.29, 1.46)
Indirect	1.01 (1.01, 1.02)	1.00 (1.00, 1.01)	1.01 (1.00, 1.01)
Proportion mediated	4.65%	1.94%	2.22%
**Midwife vs other primary attendant at birth**			
Direct	1.35 (1.32, 1.38)	1.34 (1.29, 1.40)	1.39 (1.31, 1.48)
Indirect	0.98 (0.98, 0.99)	1.00 (0.99, 1.01)	1.00 (0.99, 1.02)
Proportion mediated	0	0	0
**Induction**			
Direct	1.33 (1.31, 1.37)	1.34 (1.29, 1.40)	1.40 (1.31, 1.48)
Indirect	1.00 (1.00, 1.00)	1.00 (1.00, 1.00)	1.00 (1.00,1.00)
Proportion mediated	0.76%	0.47%	0.53%
**Overweight or Obese**			
Direct	1.27 (1.24, 1.30)	1.27 (1.22, 1.32)	1.31 (1.23, 1.39)
Indirect	1.05 (1.04, 1.05)	1.06 (1.05, 1.06)	1.07 (1.06, 1.07)
Proportion mediated	19.00%	21.13%	21.93%
**Public vs Private insurance**			
Direct	1.30 (1.27, 1.33)	1.30 (1.25, 1.35)	1.34 (1.26, 1.42)
Indirect	1.03 (1.03, 1.04)	1.03 (1.03, 1.04)	1.04 (1.03, 1.04)
Proportion mediated	12.59%	12.40%	12.52%
**Inadequate Prenatal Care**			
Direct	1.34 (1.20, 1.37)	1.34 (1.29, 1.40)	1.39 (1.31, 1.48)
Indirect	1.00 (1.00,1.00)	1.00 (1.00, 1.00)	1.00 (1.00, 1.00)
Proportion mediated	0.72%	0.98%	0.70%
**WIC**			
Direct	1.27 (1.24, 1.30)	1.28 (1.23, 1.33)	1.33 (1.25, 1.41)
Indirect	1.06 (1.06, 1.07)	1.06 (1.05, 1.06)	1.04 (1.03, 1.05)
Proportion mediated	23.49%	20.29%	14.72%
**Rural vs Urban**			
Direct	1.36 (1.32, 1.39)	1.37 (1.31, 1.42)	1.40 (1.32, 1.50)
Indirect	0.99 (0.98, 0.99)	0.98 (0.98, 0.99)	0.99 (0.99, 1.00)
Proportion mediated	0	0	0
**Any Hypertension** ^1^			
Direct	1.30 (1.27, 1.33)	1.32 (1.27, 1.37)	1.37 (1.29, 1.46)
Indirect	1.03 (1.02, 1.03)	1.02 (1.02, 1.03)	1.02 (1.01, 1.02)
Proportion mediated	9.98%	8.15%	6.29%
**Any Diabetes** ^2^			
Direct	1.34 (1.31, 1.37)	1.34 (1.29, 1.40)	1.39 (1.31, 1.48)
Indirect	1.00 (1.00, 1.00)	1.00 (1.00, 1.00)	1.00 (1.00, 1.00)
Proportion mediated	0	1.05%	0.58%

1 Any hypertension includes preeclampsia, unspecified, pregestational, and gestational hypertension

2 Any diabetes includes unspecified, pregestational, and gestational diabetes

**Table 4.** Age-adjusted mediation results for low-risk CD comparing Biracial to White individuals by ICE_Race-Income_, California 2011–2019.

	Tertile 1 (most segregated)	Tertile 2	Tertile 3 (least segregated)
**High School or Less vs Some College or Higher**			
Direct	1.14 (1.08, 1.22)	1.17 (1.07, 1.27)	1.16 (1.04, 1.29)
Indirect	1.00 (1.00, 1.01)	1.00 (1.00, 1.01)	1.01 (1.00, 1.01)
Proportion mediated	1.92%	2.37%	4.39%
**Midwife vs other primary attendant at birth**			
Direct	1.16 (1.09, 1.24)	1.20 (1.11, 1.31)	1.19 (1.07, 1.32)
Indirect	0.99 (0.98, 1.00)	0.97 (0.96, 0.99)	1.00 (0.98, 1.02)
Proportion mediated	0	0	0
**Induction**			
Direct	1.15 (1.08, 1.23)	1.18 (1.09, 1.28)	1.18 (1.06, 1.31)
Indirect	1.00 (1.00, 1.00)	1.00 (1.00, 1.00)	1.00 (1.00,1.01)
Proportion mediated	1.46%	0.50%	1.66%
**Overweight or Obese**			
Direct	1.11 (1.04, 1.18)	1.12 (1.03, 1.21)	1.13 (1.02, 1.26)
Indirect	1.05 (1.04, 1.05)	1.06 (1.05, 1.07)	1.04 (1.03, 1.05)
Proportion mediated	27.49%	35.28%	26.19%
**Public vs Private insurance**			
Direct	1.13 (1.06, 1.21)	1.15 (1.06, 1.25)	1.15 (1.03, 1.28)
Indirect	1.02 (1.01, 1.02)	1.02 (1.01, 1.02)	1.02 (1.01, 1.02)
Proportion mediated	11.71%	10.15%	11.48%
**Inadequate Prenatal Care**			
Direct	1.15 (1.08, 1.23)	1.18 (1.09, 1.28)	1.18 (1.06, 1.32)
Indirect	1.00 (1.00,1.00)	1.00 (1.00, 1.00)	1.00 (1.00, 1.00)
Proportion mediated	0.68%	0.63%	0.58%
**WIC**			
Direct	1.11 (1.04, 1.18)	1.14 (1.05, 1.24)	1.15 (1.03, 1.27)
Indirect	1.05 (1.04, 1.05)	1.03 (1.02, 1.03)	1.02 (1.01, 1.02)
Proportion mediated	31.68%	17.68%	13.10%
**Rural vs Urban**			
Direct	1.16 (1.10, 1.24)	1.19 (1.10, 1.29)	1.19 (1.07, 1.32)
Indirect	0.99 (0.99, 1.00)	0.99 (0.99, 0.99)	1.00 (0.99, 1.00)
Proportion mediated	0	0	0
**Any Hypertension** ^1^			
Direct	1.14 (1.07, 1.21)	1.17 (1.08, 1.27)	1.17 (1.05, 1.31)
Indirect	1.01 (1.01, 1.02)	1.01 (1.00, 1.02)	1.01 (1.00, 1.02)
Proportion mediated	9.78%	5.95%	5.71%
**Any Diabetes** ^2^			
Direct	1.15 (1.08, 1.23)	1.18 (1.09, 1.29)	1.18 (1.06, 1.32)
Indirect	1.00 (0.99, 1.00)	1.00 (0.99, 1.00)	1.00 (1.00, 1.00)
Proportion mediated	0	0	0

1 Any hypertension includes preeclampsia, unspecified, pregestational, and gestational hypertension

2 Any diabetes includes unspecified, pregestational, and gestational diabetes

## Data Availability

The data that support the findings of this study are available from the California Department of Public Health (CDPH). Restrictions apply to the availability of these data, which were used under license for this study. Authors do not have permission to share data. We direct researchers to the CDPH Center for Health Statistics and Information, and the California Department of Health Care Access and Information for information on requesting and accessing California state data.

## Abbreviations

ACS: American Community Survey
AGA: Appropriate for Gestational Age
BMI: Body Mass Index
CD: Cesarean Delivery
HCAI: Department of Health Care Access and Information
ICD: International Classification of Diseases
ICE: Index of the Concentrations at the Extremes
LGA: Large for Gestational Age
NTSV: Nulliparous, Term, Singleton, Vertex
SGA: Small for Gestational Age
SOMI: Study of Outcomes in Mothers and Infants
UCSD: University of California, San Diego
WIC: Special Supplemental Nutrition Program for Women, Infants, and Children
